# Exploration of SUSD3 in pan-cancer: studying its role, predictive analysis, and biological significance in various malignant tumors in humans

**DOI:** 10.3389/fimmu.2025.1521965

**Published:** 2025-03-21

**Authors:** Fei Zhong, Shining Mao, Shuangfu Peng, Jiaqi Li, YanTeng Xie, Ziqian Xia, Chao Chen, Aijun Sun, Shasha Zhang, Shiyan Wang

**Affiliations:** ^1^ Department of Laboratory Medicine, The Affiliated Huai’an Hospital of Xuzhou Medical University, The Second People’s Hospital of Huai’an, Huai’an, Jiangsu, China; ^2^ Faculty of Life Science and Food Engineering, Huaiyin Institute of Technology, Huaian, Jiangsu, China; ^3^ Key Laboratory of Systems Biomedicine, Shanghai Center for Systems Biomedicine, Shanghai Jiao Tong University, Shanghai, China

**Keywords:** SUSD3, pan-cancer, prognostic biomarker, immune-related factors, immunotherapy

## Abstract

**Background:**

The SUSD3 protein, marked by the Sushi domain, plays a key role in cancer progression, with its expression linked to tumor advancement and patient prognosis. Altered SUSD3 levels could serve as a predictive biomarker for cancer progression. Recognized as a novel susceptibility marker, SUSD3 presents a promising target for antibody-based therapies, offering a potential approach for the prevention, diagnosis, and treatment of breast cancer.

**Methods:**

Using the HPA and GeneMANIA platforms, the distribution of SUSD3 protein across tissues was analyzed, while expression levels in tumor and healthy tissues were compared using The Cancer Genome Atlas data. The TISCH and STOmics DB databases facilitated the mapping of SUSD3 expression in different cell types and its spatial relationship with cancer markers. Univariate Cox regression assessed the prognostic significance of SUSD3 expression in various cancers. Genomic alterations of SUSD3 were explored through the cBioPortal database. The potential of SUSD3 as a predictor of immunotherapy response was investigated using TIMER2.0, and GSEA/GSVA identified related biological pathways. Drugs targeting SUSD3 were identified through CellMiner, CTRP, and GDSC databases, complemented by molecular docking studies. *In vitro* experiments demonstrated that SUSD3 knockdown in breast cancer cell lines significantly reduced proliferation and migration.

**Results:**

SUSD3 expression variations in pan-cancer cohorts are closely linked to the prognosis of various malignancies. In the tumor microenvironment (TME), SUSD3 is predominantly expressed in monocytes/macrophages and CD4+ T cells. Research indicates a strong correlation between SUSD3 expression and key cancer immunotherapy biomarkers, immune cell infiltration, and immunomodulatory factors. To explore its immune regulatory role, StromalScore, ImmuneScore, ESTIMATE, and Immune Infiltration metrics were employed. Molecular docking studies revealed that selumetinib inhibits tumor cell proliferation. Finally, SUSD3 knockdown reduced cancer cell proliferation and migration.

**Conclusion:**

These findings provide valuable insights and establish a foundation for further exploration of SUSD3’s role in pan-carcinomas. Additionally, they offer novel perspectives and potential targets for the development of innovative therapeutic strategies in cancer treatment.

## 1 Introduction

Cancer remains one of the most formidable public health threats to human life and well-being. As a leading cause of mortality, it also constitutes a significant barrier to the extension of life expectancy worldwide (1). The Global Report on Cancer Statistics 2022, published on April 4, 2024, provides an updated overview of the incidence, mortality, and disease burden across 36 cancer types in 185 countries, offering insights into their distribution by gender and age group (2). Notably, the global impact of breast cancer on women’s health is particularly profound, with the incidence of this malignancy ranking first among women (3). The onset of many cancers is intricately linked to genetic mutations and deletions. Under normal circumstances, human growth and development are meticulously regulated by genes. However, genetic aberrations can disrupt this finely tuned process, triggering uncontrolled cellular proliferation and ultimately facilitating the emergence of cancer. Consequently, a deeper understanding of the molecular mechanisms underlying cancer, coupled with comprehensive cancer-wide expression analyses, is crucial for advancing diagnostic and therapeutic strategies. In recent years, large-scale initiatives such as The Cancer Genome Atlas (TCGA) have amassed a wealth of data, providing invaluable resources for future cancer research (4, 5). By systematically integrating and analyzing these datasets, researchers can explore the functional roles and prognostic significance of cancer-associated genes, thereby laying a solid foundation for the resolution and treatment of this pervasive disease.

The SUSD protein family, which includes SUSD2, SUSD3, SUSD4, and SUSD6, is characterized by the presence of Sushi domains. The expression levels of these proteins are closely linked to the prognosis of various cancers. For instance, SUSD2, a Sushi-containing protein (6), is primarily involved in a wide range of physiological and pathological processes beyond complement regulation, exhibiting distinct regulatory effects across different tumor types (7). SUSD4, which acts by inhibiting the complement system, plays a significant role in immune regulation (8). SUSD6, a negative regulator of MHC-I, is implicated in immunomodulation (9). SUSD3, a novel gene within the E2/ER signaling pathway, is particularly associated with immune modulation and has been identified as a potential biomarker for the therapeutic sensitivity of aromatase inhibitors (AIs) in breast cancer treatment (10). Despite the growing body of research, the full extent of the SUSD family’s role in tumors remains underexplored. Therefore, a comprehensive analysis of its functions, regulatory mechanisms, and structural and spatial distribution is essential. Such studies may offer new insights into the development of innovative cancer therapies (11).

With the growing body of research elucidating the relationship between the SUSD protein family and cancer, interest in this family of proteins has intensified. In order to unravel the pathogenesis of cancer and identify potential therapeutic targets, a thorough and in-depth exploration of the target genes is essential to better understand their functional roles and regulatory mechanisms. SUSD3, also known as MGC26847, is a Sushi-containing protein 3, characterized as a cell surface protein with extracellular, transmembrane, and cytoplasmic domains. It is notably overexpressed in estrogen-sensitive tissues, including the liver, breast tissue, myometrium, endometrium, and ovaries, with particularly high expression in breast cancer (12). Previous studies have highlighted SUSD3 as a critical mediator of estrogen signaling in breast cancer cells, suggesting its involvement in estrogen-dependent metastatic processes. It has been proposed as a potential biomarker for predicting both the occurrence and prognosis of breast cancer, particularly in relation to estrogen receptor (ER) and progesterone receptor (PR) status (13). Additionally, SUSD3 has been shown to influence cell growth and migration, thereby contributing to tumor progression (14, 15). As research continues to evolve, SUSD3 is poised to emerge as a promising target for the diagnosis and treatment of various cancer types, offering novel avenues for therapeutic intervention. However, to date, investigations into the role of SUSD3 have primarily focused on a single cancer type, and its broader implications across other malignancies remain inadequately explored.

This article offers a comprehensive analysis and discussion of the role of SUSD3 across various cancer types. The expression levels of SUSD3 in different tissues were assessed using the TIMER database, followed by an exploration of its functional role in cancer, including its interactions and co-expression with other proteins. Additionally, the expression and prognostic significance of SUSD3 across multiple tumors were investigated through the use of databases such as TISCH, STOmics DB, TCGA, cBioPortal, GSCA, and others. The relationship between the SUSD3 gene and key immunomodulators, tumor mutational burden (TMB), and microsatellite instability (MSI) was also explored, further establishing SUSD3 as a promising biomarker for cancer immunotherapy. The study examined the correlation between SUSD3 expression and immune cell infiltration, alongside its association with immunomodulatory genes, to better understand its biological relevance in tumor biology. Moreover, molecular docking analysis was conducted on potential therapeutic agents sourced from the CellMiner, CTRP, and GDSC databases, testing the sensitivity of SUSD3 to various anticancer drugs. The findings underscore the pivotal role of SUSD3 in cancer research and development, presenting new targets and therapeutic strategies for cancer treatment. Additionally, SUSD3 emerges as a reliable prognostic marker for numerous cancers, opening new avenues for further investigation into its clinical application in oncology.

## 2 Methods

### 2.1 Data access and processing

Initially, a bubble map illustrating diseases associated with SUSD3 was generated using the openTargetWeb tool (URL: https://platform.opentargets.org/). The subcellular localization of SUSD3 was subsequently determined through data from the Human Protein Atlas (HPA) database (URL: https://www.proteinatlas.org/). Following this, pan-cancer data downloaded from the TIMER database were utilized to analyze the differential expression of SUSD3 and its RNA sequencing data across various cancer types. These datasets were then processed in a unified manner using the Toil pipeline to ensure consistency across all data points (16). To further investigate the expression of SUSD3 across different organs, with 33 distinct expression levels, the Cancer Cell Line Encyclopedia (CCLE) database (URL: https://portals.broadinstitute.org/ccle/) was leveraged to compute cancer cell coefficients. Simultaneously, the GeneMANIA tool (URL: http://www.genemania.org) was employed to construct a protein-protein interaction (PPI) network (17), providing crucial information on the interactions of the SUSD3 protein. This network aided in formulating hypotheses regarding gene functions and identifying genes with comparable roles, particularly in areas such as physical interactions, immune-related pathways, prediction models, colocalization, genetic interactions, and shared protein domains (18). Furthermore, data from two cohorts - GSE120575 and GSE103322 (URL: https://www.ncbi.nlm.nih.gov/geo/), were incorporated for further analysis. The GSE103322 dataset consisted of 5,902 single cells from 18 patients with oral tumors (HINSCs), while the GSE120575 dataset contained melanoma (SKCM) samples from 48 patients treated with checkpoint inhibitors, resulting in the acquisition of 16,291 immune cells from their tumor samples. Specific details on cancer abbreviations are provided in **Supplementary Table S1**.

### 2.2 Single-cell analysis and spatial transcriptome analysis of SUSD3

Relevant single-cell analyses were conducted utilizing the TISCH network tool (19). The analytical parameters included the gene SUSD3, major lineage (cell type annotation), and all cancer types. To quantify and visualize the expression of SUSD3 across different cell types, heatmaps, scatter plots, and violin plots were employed. Additionally, the spatial distribution of SUSD3 was examined using the STOmics DB transcription database (https://db.cngb.org/stomics/) (20). The analysis revealed that SUSD3 exhibited significant spatial overlap not only with M2 macrophage markers CD163 and CD68, but also with the tumor cell marker CD4+ T cells, suggesting a complex role in the TME.

### 2.3 Predictive analysis of SUSD3 in patients with pan-cancer

Our findings encompass four distinct types of prognostic data: overall survival (OS), disease-specific survival (DSS), disease-free interval (DFI), and progression-free survival (PFS). To assess the prognostic impact of SUSD3 expression across various cancer types, we analyzed data from the TCGA database using Cox regression analysis, facilitated by the PanCanSurvPlot web platform (URL: https://smuonco.shinyapps.io/PanCanSurvPlot/). Additionally, the IlluminaHiSeq platform was employed to determine the optimal cut-point grouping method for each cancer type. The hazard ratio (HR) was then calculated and visualized as a “woodland” plot using the R package, with a 95% confidence interval (95% CI) to assess the statistical significance of the results.

### 2.4 Alterations in the cancer-associated genome of SUSD3

Genomic alterations across four distinct types were analyzed in tumors using the cancer type summary module from the cBioPortal platform (URL: https://www.cbioportal.org/) (21). Subsequently, the GSCA database (22) (URL: http://bioinfo.life.hust.edu.cn/GSCA) was utilized to assess the differential methylation levels of the SUSD3 gene across various cancer tissues, as well as the correlation between SUSD3 mRNA expression and its methylation status. Survival differences between these factors were also compared. Spearman correlation analysis was conducted to explore the relationship between SUSD3 copy number variation (CNV) and mRNA expression across pan-cancer datasets, with p-values adjusted for false discovery rate (FDR). Finally, a time-series analysis was performed to minimize statistical discrepancies in OS, DSS, and PFS.

### 2.5 Predictive analysis of immunotherapy

Somatic mutation data were retrieved from the TCGA database (https://tcga.xenahubs.net) and subsequently analyzed using the R package “maftools” to calculate the TMB and MSI for each sample. Spearman correlation analysis was performed to assess the statistical relationships between SUSD3 and known immunotherapy biomarkers, TMB, MSI, and other established immune checkpoint genes across various cancers. The results were visualized using radar plots generated by the “ggradar” package in R. Additionally, the optimal cutoff values were determined using the R package “Survival”, and the resulting two treatment cohorts were utilized to compute survival rates and analyze immune responses. Further investigation was conducted to explore the relationship between MMR genes (including MLH1, MSH2, MSH6, PMS2, and EPCAM) and SUSD3 expression. The findings were presented in heatmaps generated using the “tidyverse” and “ggnewscale” R packages.

### 2.6 Effect of SUSD3 expression on immunity

First, the correlation between SUSD3 gene expression and the TME was analyzed using the Illumina platform. The “limma” and “estimate” R packages were then employed to comprehensively evaluate the stromal, immune, and ESTIMATE scores across different tumor types. Next, Spearman correlation analysis was conducted using data from the TCGA and TIMER 2.0 databases to explore the relationship between SUSD3 expression and immune cell infiltration, with an emphasis on potential interactions between these factors. Additionally, 150 immune-related genes, including those encoding major histocompatibility complex (MHC) molecules, immune suppressive factors, chemokine receptors, immune activation factors, and chemokine proteins, were downloaded from the TISDB database. The relationships between these immune-related genes and SUSD3 were analyzed using the “limma”, “pheatmap”, and “ggplot2” R packages (23).

### 2.7 Significance of SUSD3 in biology

GSEA and GSVA were performed on SUSD3 using the “tidyverse”, “limma”, “org.Hs.eg.db”, “gseaplot2”, and “clusterProfiler” R packages (3) to explore its biological significance in various tumors. Based on the median expression levels of SUSD3 across different cancer types, normalized enrichment scores were calculated, and samples were grouped accordingly. The differential gene expression between high and low expression groups was compared by assessing the FDR. To further investigate the biological relevance of SUSD3 in cancer, the samples were classified into high and low expression groups, and GSVA analysis was conducted using the GSVA, “ggprism”, “GSEABase”, “ggthemes”, “BiocParallel”, “tidyverse”, and “clusterProfiler” R packages. This approach enabled a comprehensive evaluation of the differential enrichment of biological pathways and gene sets associated with SUSD3 expression in the tumor context.

### 2.8 Drug sensitivity and molecular docking of SUSD3

To explore the potential association between SUSD3 and drug response, cancer researchers utilized several databases, including the CellMiner database (URL: http://discover.nci.nih.gov/cellminer/) (24), the CTRP database (URL: http://portals.broadinstitute.org/ctrp/), and the GDSC database (URL: https://www.cancerrxgene.org/). These resources were leveraged to conduct anticancer drug screening using the NCI-60 cancer cell line panel, and the data from this cell line collection were compiled and downloaded via CellMiner. The analysis and filtering of FDA-approved or clinical trial-stage drug data were conducted using the “limma” and “Impute” R packages. Data with over 80% missing values were discarded, and the remaining missing data were imputed using the “Impute” package. Data visualization was performed using the “ggplot2” and “pub” packages, with statistical significance set at a p-value less than 0.05. A drug of interest, selumetinib, was selected from the CellMiner, CTRP, and GDSC databases. Molecular docking analysis of selumetinib and the SUSD3 protein was performed using AutoDock4 software (25), which provided insights into the binding modes and binding energies between selumetinib and the SUSD3 protein. Subsequently, the molecular structure of selumetinib was obtained from the PubChem database (URL: https://pubchem.ncbi.nlm.nih.gov/), and the 3D structure of the SUSD3 protein was retrieved from AlphaFold (URL: https://alphafold.ebi.ac.uk/) (26, 27). Visualization and further analysis of the docking models were carried out using PyMOL software.

### 2.9 Culture of cells

The human breast cancer cell line MCF-7 was purchased from the Chinese Academy of Sciences Cell Bank (Shanghai, China). Cells were cultured in a medium supplemented with 10% fetal bovine serum (FBS; Procell) under standard conditions of 37°C and 5% CO2, with regular monitoring for mycoplasma contamination. The human SUSD3 siRNA and negative control siRNA used in the experiments were provided by GenePharma (Shanghai, China). Following the manufacturer’s instructions, siRNA transfection was carried out using the Lipofectamine 3000 reagent (Invitrogen, CA, USA). The specific sequences of the siRNAs used are provided in **Supplementary Table S2**.

### 2.10 Reverse transcription-quantitative polymerase chain reaction (RT-qPCR)

Total RNA was extracted using TRIzol reagent (Takara Bio, Kusatsu, Japan), and RNA concentration and purity were measured using the NanoDrop 2000 system (Thermo Scientific). For reverse transcription PCR (RT-PCR), PrimeScript™ RT Master Mix (Takara, RR036A) was used, while real-time quantitative PCR (qPCR) was performed with Sybr^®^Ex Taq™II (Takara, RR820A). Gene expression analysis was conducted using the 2^-ΔΔCt^ method, with ACTB serving as the internal reference gene. The primer sequences for RT-qPCR are provided in **Supplementary Table S3**.

### 2.11 Determination of cell viability

Cell viability was accurately measured using the CCK-8 kit (GK10001, GLPBIO, Montclair, California, USA). Cells were seeded in 96-well plates at a density of 2×10³ per well according to the manufacturer’s instructions. After 24 or 48 hours of incubation, 10 μl of CCK-8 reagent was added to each well. Following an additional 2-hour incubation, absorbance at 450 nm was measured using a microplate reader to obtain the results, which were subsequently analyzed.

### 2.12 Formation of colonies

The MCF-7 cell line was seeded at a density of 1,000 cells per well on a 6-well plate, with three replicates for each condition. The medium was replaced with fresh medium every three days. One week later, the cells were fixed with methanol and stained with 0.5% crystal violet. After allowing sufficient time for staining, the cells were photographed, and the number of cloned cells was quantified using ImageJ software.

### 2.13 Cell scratch wound healing assay

For the wound healing assay, cells transfected with the specified siRNA were plated in 6-well plates at a density of 1 × 10^5^ cells per well, with 2% FBS added to the culture medium to minimize the influence of cell proliferation on the results. A scratch was introduced into the cell monolayer using a micropipette tip. Images of the wound area were captured at 0 and 48 hours using a Nikon Ti-E inverted microscope (Nikon Instruments, Florence, Italy). The wound area at each time point was measured and analyzed with ImageJ software, with the measurements at each time point normalized to the specific moment of the initial wound area (T0).

### 2.14 Statistical analysis

The dataset was first processed by removing missing values and duplicate results, followed by log2 (TPM + 1) transformation of the TPM values. The Wilcoxon rank-sum test was then applied to compare the expression of SUSD3 between normal and tumor tissues to determine statistical significance. Based on the CCLE database, the Kruskal-Wallis test was used to analyze the expression of SUSD3. The paired or unpaired nature of the samples determined whether a paired t-test or an unpaired t-test was employed to compare SUSD3 expression levels between different tissues or between tumor and normal tissues. A significance level of p < 0.05 was used. All analyses were performed using R software (version 4.4.0; https://www.R-project.org).

## 3 Results

### 3.1 Differential expression and related genes of SUSD3 in pan carcinoma

**Figure 1:**
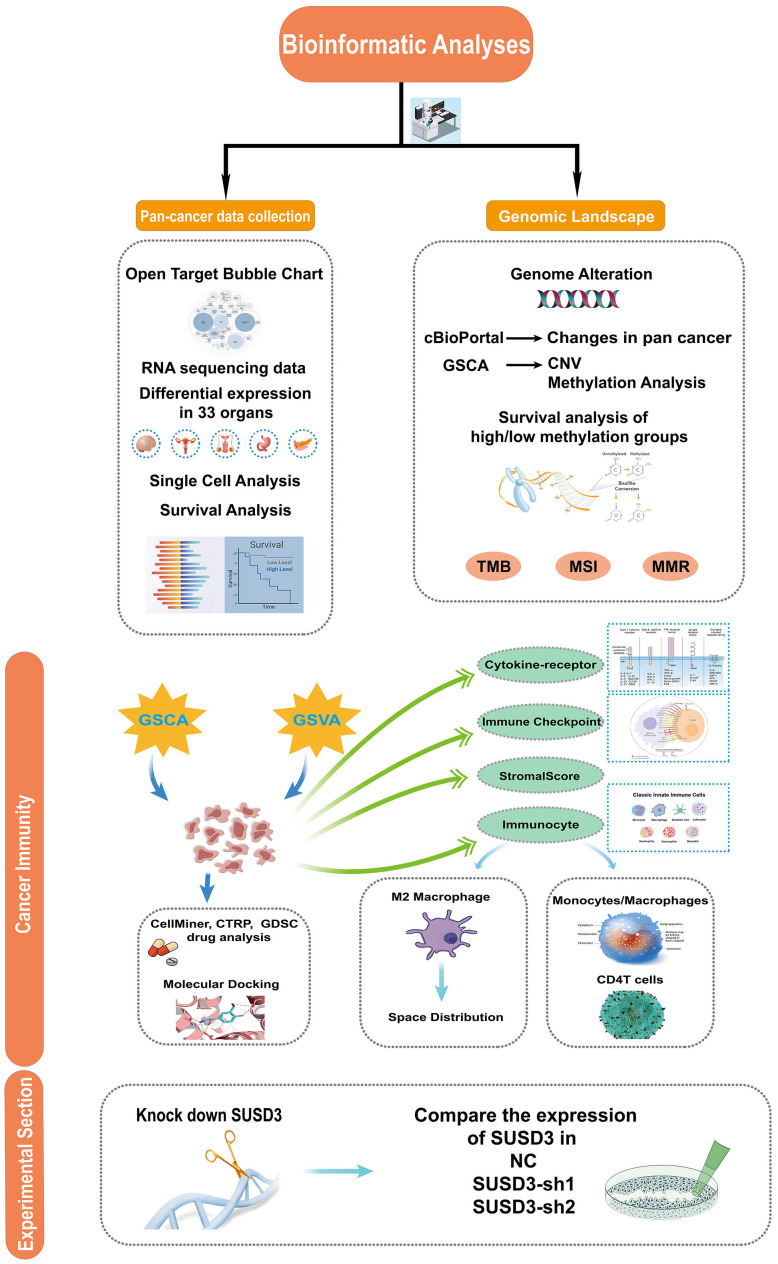
The study design flowchart outlines a systematic exploration of the target gene SUSD3 across a wide spectrum of cancers. Initially, the expression of SUSD3 in pan-cancer was assessed, followed by an analysis of its association with various cancer types. The study compares expression disparities of SUSD3 across 33 malignant and non-malignant tissues, as well as different cellular contexts. Subsequent analyses involved single-cell transcriptomic and prognostic evaluations. A comprehensive investigation of the genomic landscape was conducted, focusing on genomic instability, utilizing data from the cBioPortal and GSCA databases to analyze pan-cancer alterations, including CNVs and DNA methylation patterns. Further exploration was undertaken to examine the correlation between SUSD3 expression and TMB, MSI, and MMR status. To elucidate the functional role of SUSD3 in oncogenesis, the study delved into its involvement in immune modulation, analyzing sequence function enrichment, immune checkpoint dynamics, cytokine receptor interactions, and immune cell infiltrates. Finally, the potential therapeutic implications of SUSD3 were explored through predictions of chemotherapy response, drug sensitivity profiling, and related experimental assessments.

**Figure 2:**
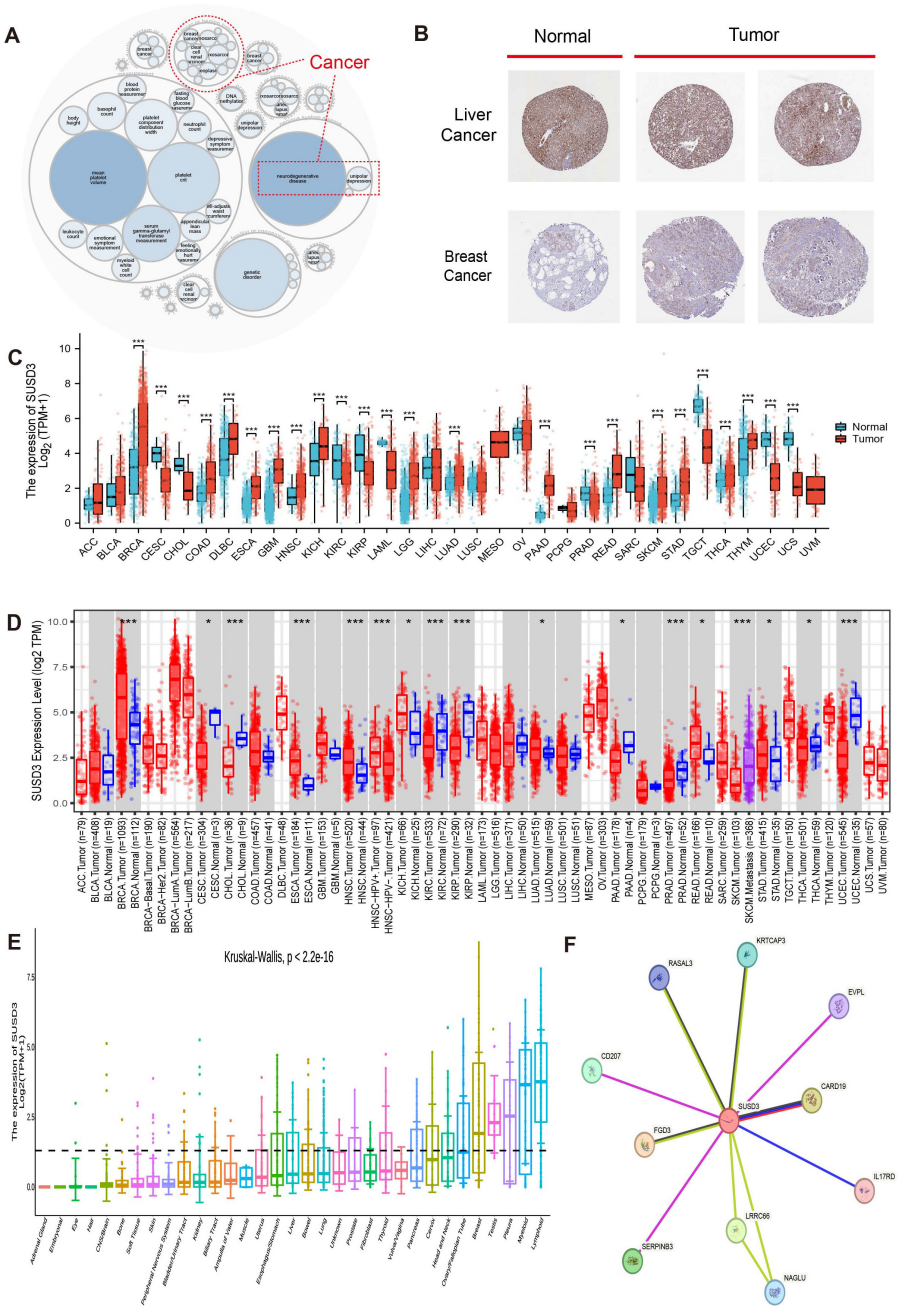
Differential expression and related genes of SUSD3 in pancarcinoma. **(A)** Analysis of SUSD3-related diseases on the openTargetWeb tool. The red dashed lines represent SUSD3-associated cancers. **(B)** SUSD3 protein expression in IHC images of the normal group (left) and tumor group (right). **(C)** Pan-cancer data downloaded from the TIMER database were used to analyze SUSD3 expression differences, and RNA sequencing data were analyzed. Statistical significance markers were *p < 0.05, **p < 0.01, ***p < 0.001. **(D)** The expression level of SUSD3mRNA in pancarcinoma and its corresponding control tissues was analyzed by TIMER2.0. (Statistical significance was marked as *p < 0.05, **p < 0.01, ***p < 0.001). **(E)** The Kruskal-Wallis map was used to observe the difference in SUSD3 expression in 33 different organs. **(F)** PPI network analysis of SUSD3 binding partners.

**Figure 1** illustrates the overall scope of the study. The diseases associated with SUSD3 were explored through the OpenTarget platform, where a bubble plot revealed a significant correlation between SUSD3 and various malignancies, including breast cancer and clear cell renal carcinoma, among others (**Figure 2A**). Following this, immunohistochemical (IHC) analysis was conducted to examine the differential expression of SUSD3 protein between cancerous and normal tissues. Notably, the expression of SUSD3 was markedly elevated in breast cancer compared to normal breast tissue (**Figure 2B**). To further investigate the variation in SUSD3 expression across different tumor types, RNA sequencing data were analyzed via the TIMER database. The findings demonstrated a pronounced upregulation of SUSD3 in several cancers, including BRCA, COAD, LGG, PAAD, LUAD, READ, and STAD, in comparison to normal tissue. Conversely, SUSD3 expression was significantly reduced in CESC, CHOL, TGCT, and UCEC (**Figure 2C**). To corroborate and delve deeper into the differences in SUSD3 mRNA expression between pancreatic cancer and normal tissues, an analysis using TIMER2.0 was performed. The results confirmed an increased expression of SUSD3 across various malignant tissues, notably in BRCA and ESCA (**Figure 2D**). Further exploration of SUSD3 expression in diverse tissues was conducted by collecting samples from 33 distinct organs to assess potential expression variations. High levels of SUSD3 expression were observed in the mammary glands, testes, pleura, bone marrow, and lymphoid organs (**Figure 2E**). To investigate the functional role of SUSD3 in cancer progression and its associated protein interactions, a PPI network was constructed using the STRING tool. The top ten proteins that were closely linked to SUSD3 were identified: KRTCAP3, EVPL, CARD19, IL17RD, LRRC66, NAGLU, SERPINB3, FGD3, CD207, and RASAL3 (**Figure 2F**). These genes exhibit a strong correlation with SUSD3 and may be pivotal in the pathogenesis of cancer.

### 3.2 Single-cell analysis of SUSD3 in various cancers

**Figure 3:**
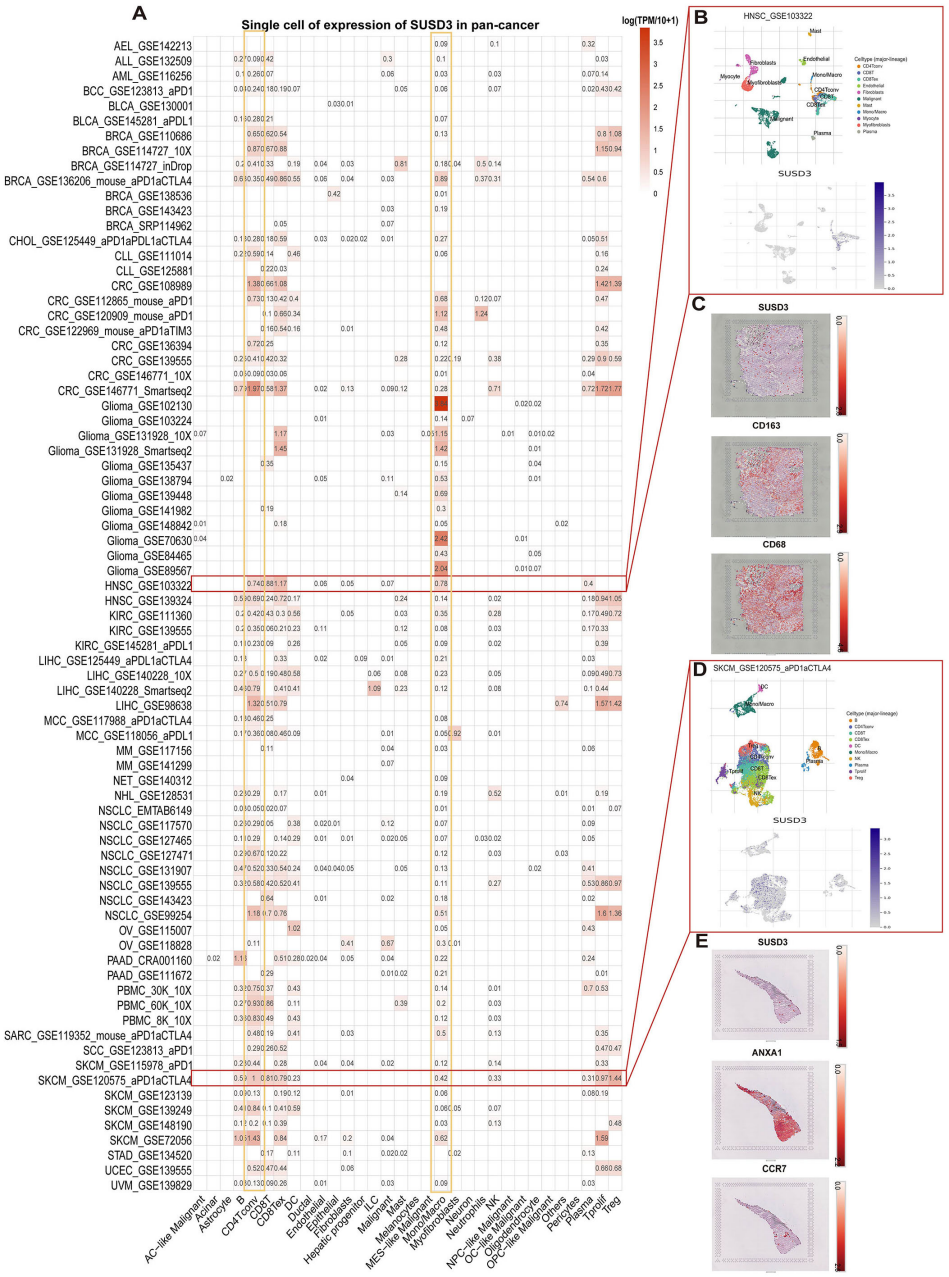
Single-cell analysis of SUSD3 in various cancers. **(A)** SUSD3 expression summary of 32 cell types from 77 single-cell datasets. **(B)** The scatter plot shows the distribution of 10 different cell types in the GSE103322HINSC dataset, as well as SUSD3 expression levels of cells in the GSE103322 dataset. **(C)** Spatial transcription slices showed the spatial expression of SUSD3, CD68, and CD163 markers, and the color of the dots indicated the expression level of the markers. **(D)** The scatter plot shows the distribution of 10 different cell types in the GSE120575 dataset, as well as SUSD3 expression levels of cells in the GSE120575 dataset. **(E)** Spatial transcription slices showed the spatial expression of SUSD3, ANXA1, and CCR7 markers, and the color of the dots indicated the expression level of the markers.

Breaking the physical barrier limitation of the TME could further optimize the existing tumor treatment protocols. To investigate the primary cell types expressing SUSD3 within the TME, a comprehensive single-cell analysis was performed on a dataset comprising 77 cancer samples. The expression levels of SUSD3 across 32 distinct cell types, including immune, stromal, malignant, and functional cells, were assessed using the TISCH network tool. The results indicated that SUSD3 was predominantly expressed in immune cells, with a notable enrichment in monocytes/macrophages and CD4+ T cells (**Figure 3A**). In a specific dataset (GSE103322) containing 5,902 single cells derived from 18 patients with HINSCs, SUSD3 expression was found to be particularly evident in CD4+ T conventional cells (Tconv), cytotoxic T lymphocytes, and CD8+ T-exhausted (Tex) cells within the HINSC tumor microenvironment (**Figure 3B**). Moreover, spatial transcriptional data obtained from the STOmics DB database revealed a spatial overlap between SUSD3 expression and markers of M2 macrophages, namely CD163 and CD68, within HINSC cancer tissues. This suggests co-expression of SUSD3, CD163, and CD68 in these cell types (**Figure 3C**). Furthermore, an analysis of the GSE120575 dataset, which encompasses 16,291 immune cells from tumor samples of 48 SKCM patients treated with immune checkpoint inhibitors (ICIs), demonstrated that SUSD3 was predominantly expressed in CD4+ Tconv cells, regulatory T (Treg) cells, cytotoxic T lymphocytes, and CD8+ Tex cells within the SKCM microenvironment (**Figure 3D**). Spatial distribution analysis using the STOmics DB database also showed that SUSD3 co-localized with the tumor cell marker ANXA1 and the T cell marker CCR7 in SKCM cancer tissues, indicating spatially similar distributions. It is likely that SUSD3, ANXA1, and CCR7 are co-expressed in these cell types (**Figure 3E**).

### 3.3 Prognostic analysis of SUSD3 expression levels in pan-cancer types

**Figure 4:**
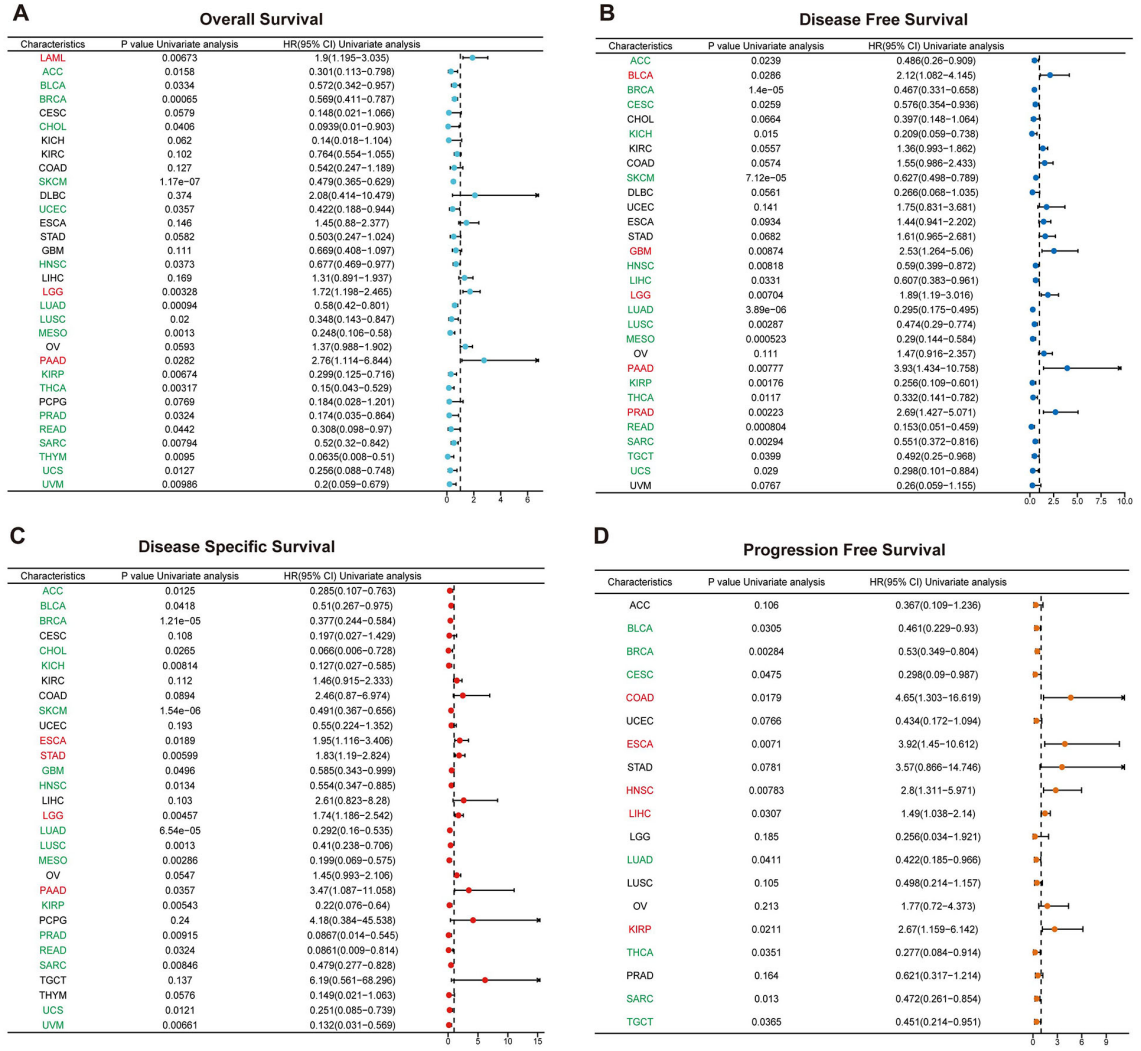
SUSD3 expression level in pan-cancer type prognosis Forest chart shows the prognostic role of SUSD3 in cancer by univariate Cox regression method. SUSD3 expression was associated with OS **(A)**, DFS **(B)**, DSS **(C)**, and PFS **(D)**. Cancer types in red indicate that SUSD3 is a statistically significant risk factor (HR value > 1) and cancer types in green indicate that SUSD3 is a statistically significant protective factor (HR value <1).

The expression levels of SUSD3 across various cancer types were analyzed using data from the TCGA database, and the association between SUSD3 expression and cancer prognosis was examined through one-way Cox regression analysis. A heatmap summarizing the prognostic analysis of SUSD3 across pan-cancer datasets revealed that SUSD3 did not correlate with the prognosis of KIRC but showed significant associations with the prognosis of most other cancer types. Using the Cox proportional hazards model, OS outcomes indicated that SUSD3 acted as a risk factor in patients with LAML, LGG, and PAAD. Conversely, SUSD3 was identified as a protective factor in patients with ACC, BLCA, BRCA, CHOL, SKCM, UCEC, HNSC, LUAD, LUSC, MESO, KIRP, THCA, PRAD, READ, SARC, THYM, UCS, and UVM (**Figure 4A**). In the DFS analysis, SUSD3 expression was positively correlated with DFS in patients with BLCA, GBM, PAAD, and PRAD. It showed a significant negative correlation with DFS in patients with ACC, BRCA, CESC, KICH, SKCM, HNSC, LIHC, LUAD, LUSC, MESO, KIRP, THCA, READ, SARC, TGCT, and UCS (**Figure 4B**). Given that OS includes deaths from non-cancer causes, a subsequent analysis focusing on the DSS was performed to better reflect the impact of cancer treatments. In the DSS analysis, SUSD3 was identified as a risk factor in patients with ESCA, STAD, LGG, and PAAD. Additionally, SUSD3 was considered a risk factor in ACC, BLCA, BRCA, CHOL, KICH, SKCM, GBM, HNSC, LUAD, LUSC, MESO, KIRP, PRAD, READ, SARC, UCS, and UVM (**Figure 4C**). Moreover, in the PFS analysis, SUSD3 was found to be a risk factor in patients with COAD, ESCA, HNSC, LIHC, and KIRP, while serving as a protective factor in patients with BLCA, BRCA, CESC, LUAD, THCA, SARC, and TGCT (**Figure 4D**).

### 3.4 The SUSD3 gene is altered in pan-cancer and is associated with genomic instability and alteration in cancer

**Figure 5:**
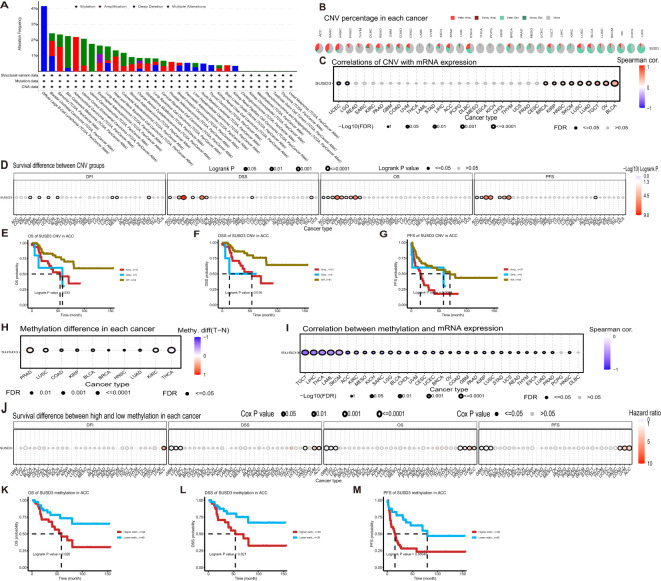
SUSD3 gene altered in pan-cancer, associated with genomic instability and alteration in cancer. **(A)** SUSD3 altered frequency analysis based on pan-cancer studies in the cBioPortal database. **(B)** The proportion of each CNV in pancarcinoma. **(C)** Spearman correlation analysis was used to investigate the relationship between SUSD3 CNV and mRNA expression. **(D)** SUSD3 CNV status was associated with OS, DSS, PFS, and DFI. **(E-G)** The Kaplan-Meier survival curve was generated by the GSCA network tool to analyze the prognostic value of SUSD3 CNVs in ACC patients. **(H)** Analysis of methylation differences between tumor and normal samples. **(I)** Spearman correlation analysis was used to study the relationship between SUSD3 methylation and mRNA expression. **(J)** SUSD3 methylation status was associated with the OS, DSS, PFS, and DFI. **(K-M)** Kaplan-Meier survival curves were generated using the GSCA network tool to describe the risk of death in ACC patients with high and low methylation status.

Cancer is inherently characterized by genomic alterations that drive its progression. To investigate whether the SUSD3 gene undergoes genomic changes across various cancer types, its alterations were analyzed using the cBioPortal database. The analysis revealed that genomic alterations in the SUSD3 gene are relatively rare across cancers, with the most common alteration occurring in DLBC, where over 4% of patients exhibit alterations, predominantly involving deep deletions (**Figure 5A**). Additionally, CNV and methylation patterns of SUSD3 were explored using the GSCA database. The CNV analysis demonstrated that the proportion of heterozygous/pure CNV varied across cancer types, with distinct colors representing different CNV categories. The results indicated that in 33 different cancers, the frequency of heterozygous deletions of SUSD3 significantly exceeded that of heterozygous amplifications (**Figure 5B**). Subsequently, Spearman correlation analyses between SUSD3 CNV and mRNA expression were performed across multiple cancers. The findings revealed a significant positive correlation between SUSD3 CNV and mRNA expression in TGCT, OV, and BLCA. Conversely, a notable negative correlation between SUSD3 CNV and mRNA expression was observed in LGG and UCEC (**Figure 5C**). Next, the samples were stratified into three groups based on CNV status—WT (wild-type), Amp (amplification), and Dele (deletion)—to examine the survival implications of SUSD3 CNV across various cancers. The results indicated that the SUSD3 CNV-high group exhibited poorer OS rates in KIRP, UCEC, KIRC, and THYM (**Figure 5D**). Further analysis revealed that patients with ACC had unfavorable prognoses across all three survival measures (**Figures 5E–G**). DNA methylation is critical in regulating gene expression and maintaining stable gene silencing, as it is intricately linked with histone modifications and chromatin structure. Thus, we performed DNA methylation analysis to compare methylation levels between normal and tumor samples (28). THCA, KIRC, LUAD, HNSC, and BRCA exhibited higher methylation levels compared to their normal counterparts, whereas PRAD and LUSC tumors showed lower methylation levels compared to normal tissues (**Figure 5H**). Spearman correlation analysis between SUSD3 methylation and mRNA expression indicated a strong association between methylation levels and mRNA expression across most cancer types, excluding PCPG and DLBC cancers. This correlation was especially pronounced in TGCT, LIHC, THCA, and LAML (**Figure 5I**). Further stratification of tumor samples into hypermethylation and hypomethylation groups revealed that patients with GBM, LGG, and LIHC tumors exhibited lower OS with increased SUSD3 methylation levels (**Figure 5J**). Conversely, higher SUSD3 methylation levels were associated with improved survival outcomes in ACC patients (**Figures 5K–M**).

### 3.5 The relationship between the SUSD3 gene and immunomodulator, TMB, and MSI

**Figure 6:**
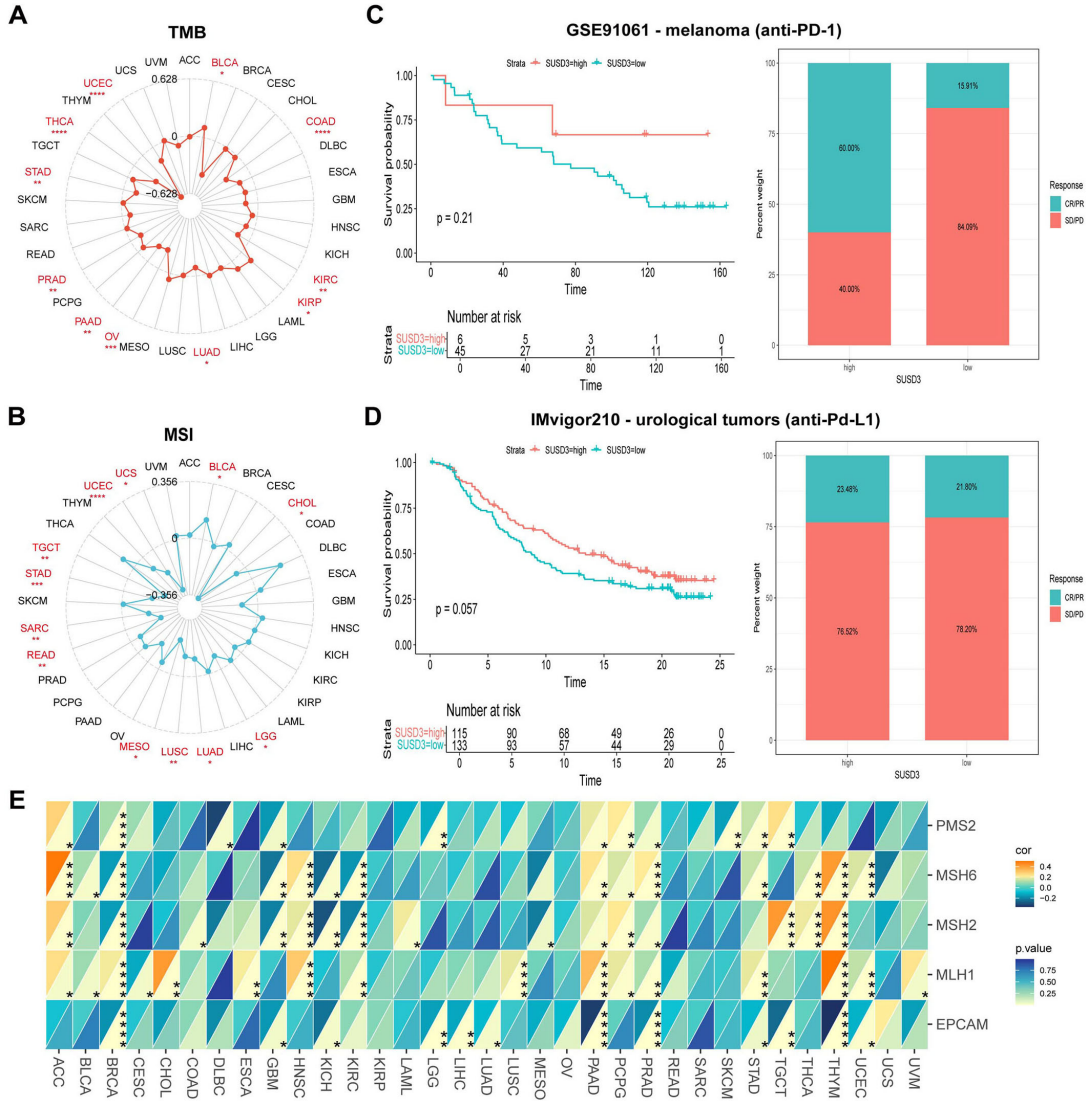
Relationship between SUSD3 gene and immunomodulators, TMB, MSI. **(A)** Correlation between SUSD3 expression and TMB in pancarcinoma. **(B)** Correlation between SUSD3 expression and MSI in pancarcinoma. **(C, D)** Proportion of patients with melanoma/urologic tumors who responded to anti-PD-1/anti-PD-L1 therapy in the GSE91061 (anti-PD-L1)/IMvigor210 (anti-PD-L1) (anti-PD-L1) and high SUSD3 patient groups in the low and high SUSD3 patient groups. **(E)** The correlation between SUSD3 expression and five MMR genes in the pan-cancer cohort was shown by heat maps. Statistical significance was marked as *p<0.05, **p<0.01, ***p<0.001, ****p<0.0001.

To further investigate the role of SUSD3 in predicting the efficacy of ICIs, the relationship between SUSD3 expression and key immunotherapy biomarkers, such as TMB and MSI, was explored. Both TMB and MSI are crucial factors in assessing the sensitivity to ICIs and have been demonstrated to significantly influence patient prognosis and treatment response (29, 30). The analysis revealed a positive correlation between SUSD3 expression and TMB in KIRP and BLCA, while a negative correlation was observed in UCEC, THCA, STAD, PRAD, PAAD, OV, LUAD, KIRC, and COAD (**Figure 6A**). Similarly, SUSD3 expression was positively correlated with MSI in BLCA but negatively correlated with MSI in CHOL, LGG, LUAD, LUSC, MESO, READ, SARC, STAD, TGCT, UCEC, and UCS (**Figure 6B**). These findings suggest that SUSD3 has the potential to predict the response to ICIs across various cancers. Further analysis was performed to assess the predictive value of SUSD3 expression in ICI treatment outcomes. In the GSE91061 melanoma cohort, patients with high SUSD3 expression exhibited significantly better survival rates compared to those with low SUSD3 expression (**Figure 6C**). Additionally, in patients with urinary tract cancers receiving anti-PD-L1 therapy, higher expression of SUSD3 was associated with improved survival and longer duration of response, as seen in the IMvigor210 cohort (**Figure 6D**). To deepen the understanding of the potential mechanisms underlying these observations, the relationship between SUSD3 expression and the expression levels of MMR genes—specifically MLH1, MSH2, MSH6, PMS2, and EPCAM—was examined. In 33 cancer types, MMR gene expression levels showed a negative correlation with SUSD3 expression in BLCA, BLCA, CESC, DLBC, KIRC, OV, READ, SARC, SCKM, and other cancers. In contrast, a positive correlation between MMR genes and SUSD3 expression was observed in ACC, CHOL, HNSC, PAAD, PCPG, and THYH (**Figure 6E**). Collectively, these results confirm the potential of SUSD3 as a predictive biomarker for immunotherapy response and underscore its promising role in cancer immunotherapy.

### 3.6 Relationship between SUSD3 gene expression and immune-related factors

**Figure 7:**
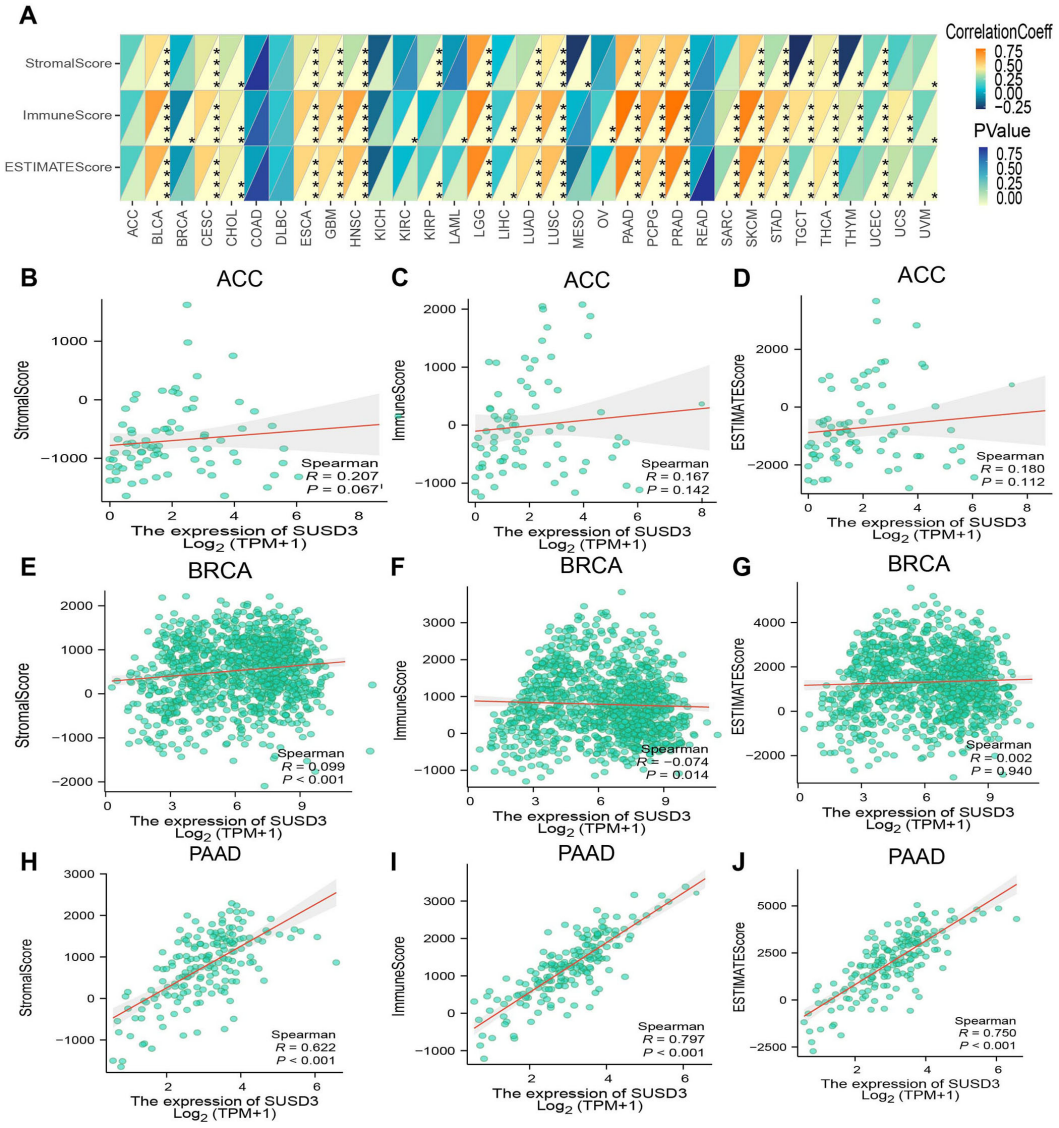
SUSD3 gene expression and immune-related factors. **(A)** Spearman Spearman analysis was used to analyze the relationship between SUSD3 expression and ImmuneScore, StromalScore, and EstimateScore. (Statistical significance was marked as *p < 0.05, **p < 0.01, ***p < 0.001, ****p < 0.0001) Relationship between the expression of SUSD3 in three cancers and ImmuneScore, StromalScore, EstimateScore: ACC **(B-D)**, BRCA **(E-G)**, PAAD **(H-J)**.

For optimal therapeutic outcomes, a comprehensive understanding of the TME is essential. The TME is a multifaceted ecosystem composed predominantly of immune cells, fibroblasts, endothelial cells, pericytes, and other specialized tissue-resident cell types (31). To explore the relationship between SUSD3 gene expression and the TME, the stromal, immune, and ESTIMATE scores across various tumor types were systematically evaluated. The findings revealed a significant positive correlation between SUSD3 expression and the StromalScore, ImmuneScore, and ESTIMATE scores in multiple cancer types, including BLCA, PAAD, PCPG, and PLAD (**Figure 7A**). Additional results for ACC, BRCA, and PAAD are presented in **Figures 7B–J**. These findings suggest that SUSD3 plays a pivotal role in modulating the TME and may be a key factor influencing tumor progression and immune responses.

### 3.7 Correlation between SUSD3 gene TIMER immune cell infiltration analysis and immunomodulatory genes

**Figure 8:**
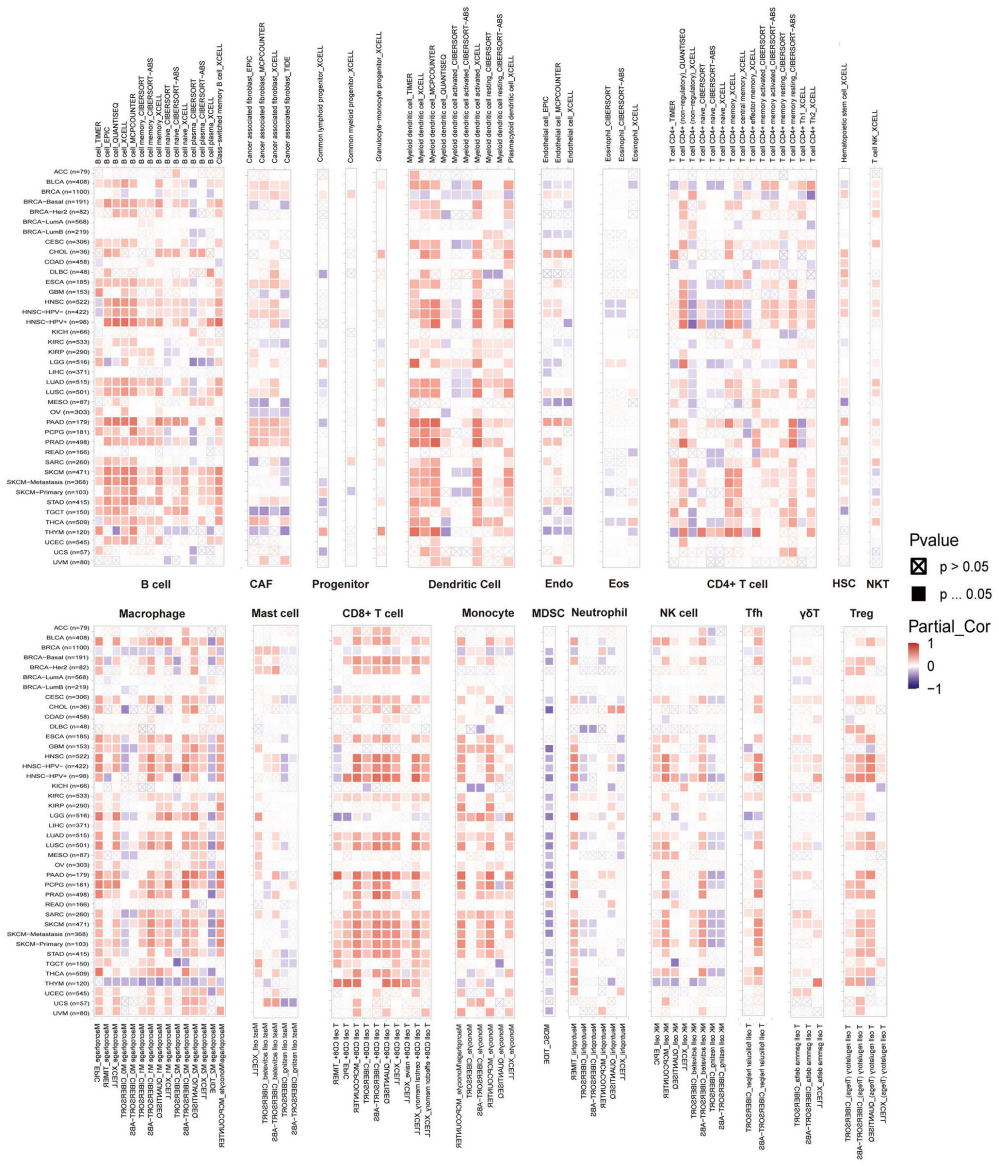
SUSD3 gene TIMER analysis of immune cell infiltration. The expression of SUSD3 in pan-cancer with B cells, CAF cells, lymphoid progenitor cells, dendritic cells, endothelial cells, eosinophils, CD4+ T cells, macrophages, mast cells, CD8+ T cells, monocytes, myeloid suppressor cells, neutrophils, NK cells, Tfh cells, γ/δ T cells, and Tregs cells were analyzed from the TIMER2.0 database. Red is positively correlated, and blue is negatively correlated.

**Figure 9:**
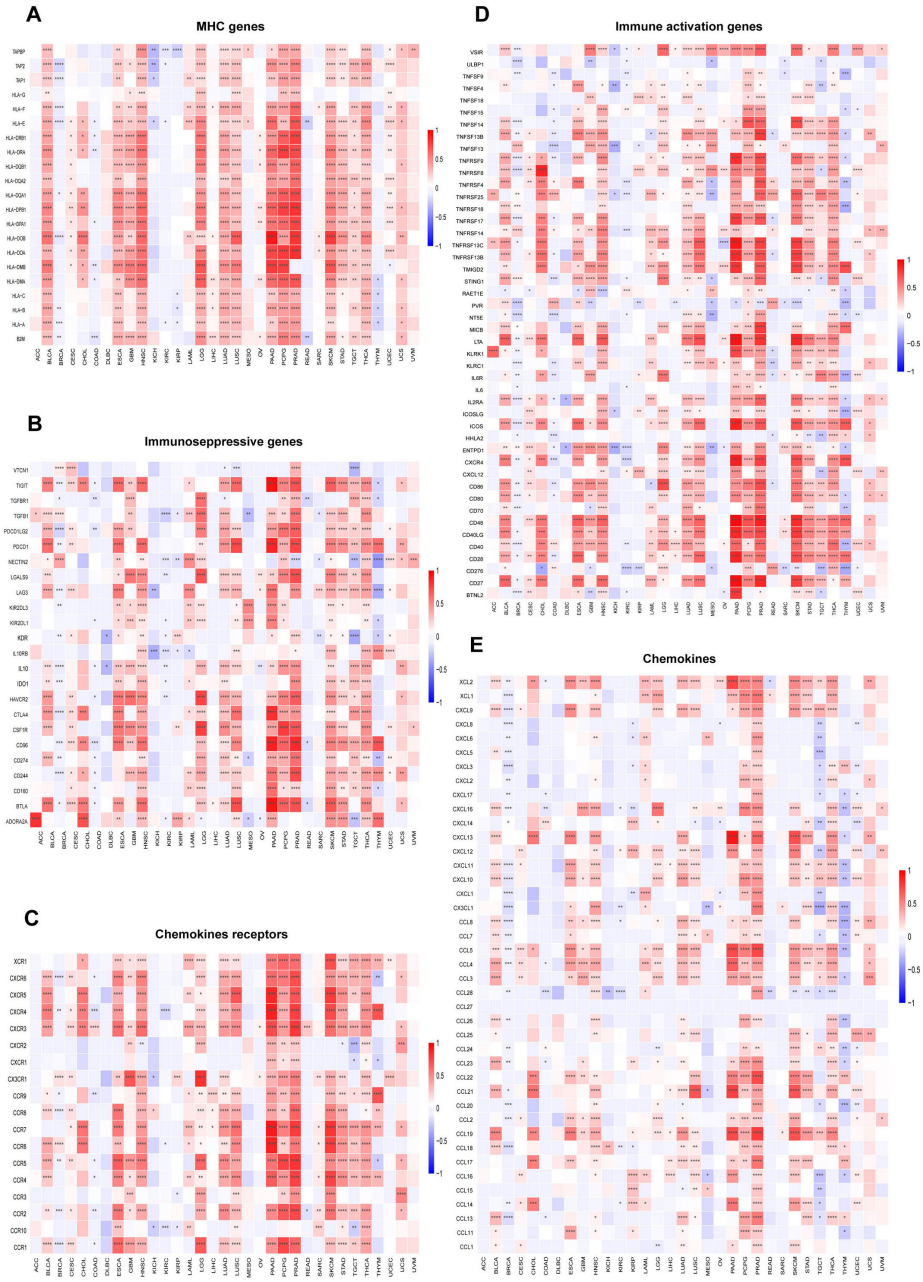
SUSD3 gene expression and immunoregulatory genes Relationship between SUSD3 expression and **(A)** MHC gene, **(B)** immunosuppressive factor, **(C)** chemokine receptor, **(D)** immune activator, **(E)** chemokine. (mark to *p < 0.05, **p < 0.01, ***p < 0.001, ****p < 0.0001).

To further elucidate the relationship between SUSD3 and cancer immunity, a detailed investigation was conducted on the correlation between SUSD3 expression and immune cell infiltration. Spearman correlation analysis was performed using pan-cancer immune cell infiltration data from the TIMER2.0 database. The analysis revealed a significant association between SUSD3 expression and the infiltration levels of various immune cell types, including B cells, CAF cells, lymphoid progenitor cells, dendritic cells, endothelial cells, eosinophils, CD4+ T cells, macrophages cells, mast cells, CD8+ T cells, monocytes, MDSCs, neutrophils, NK cells, Tfh cells, γ/δ T cells, and Tregs across a wide array of TCGA cancers. Notably, SUSD3 expression was positively correlated with the infiltration of B cells, CAF cells, dendritic cells, CD4+ T cells, macrophages, CD8+ T cells, monocytes, NK cells, and Tregs cells in most tumor types, while it exhibited a negative correlation with the infiltration of MDSCs in cancers such as CHOL, GBM, LGG, PCPG, SRC, and SKCM (**Figure 8**). Recent studies have emphasized the crucial roles of CD4+ T cells, MDSCs, neutrophils, and macrophages in cancer immunotherapy (32, 33), underlining the importance of immune cells in therapeutic responses. The findings suggest that SUSD3 may influence cancer initiation, prognosis, and therapeutic outcomes through its modulation of immune cell infiltration. In addition, the relationship between SUSD3 expression and immune response genes was examined. Analysis of pan-cancer gene expression revealed that most of the correlated genes encode MHC proteins, immunosuppressive factors, immune-activating proteins, chemokine receptors, and chemokines. As shown in the heatmap, SUSD3 exhibited a significant negative correlation with these immune response genes in BRCA, KICH, and KIRC, while it was positively correlated with the same genes in other tumor types (**Figure 9**). These results indicate that SUSD3 might play a pivotal role in shaping the immune landscape of tumors and could be a valuable target for cancer immunotherapy.

### 3.8 Biological implications for the expression of SUSD3 in tumors

**Figure 10:**
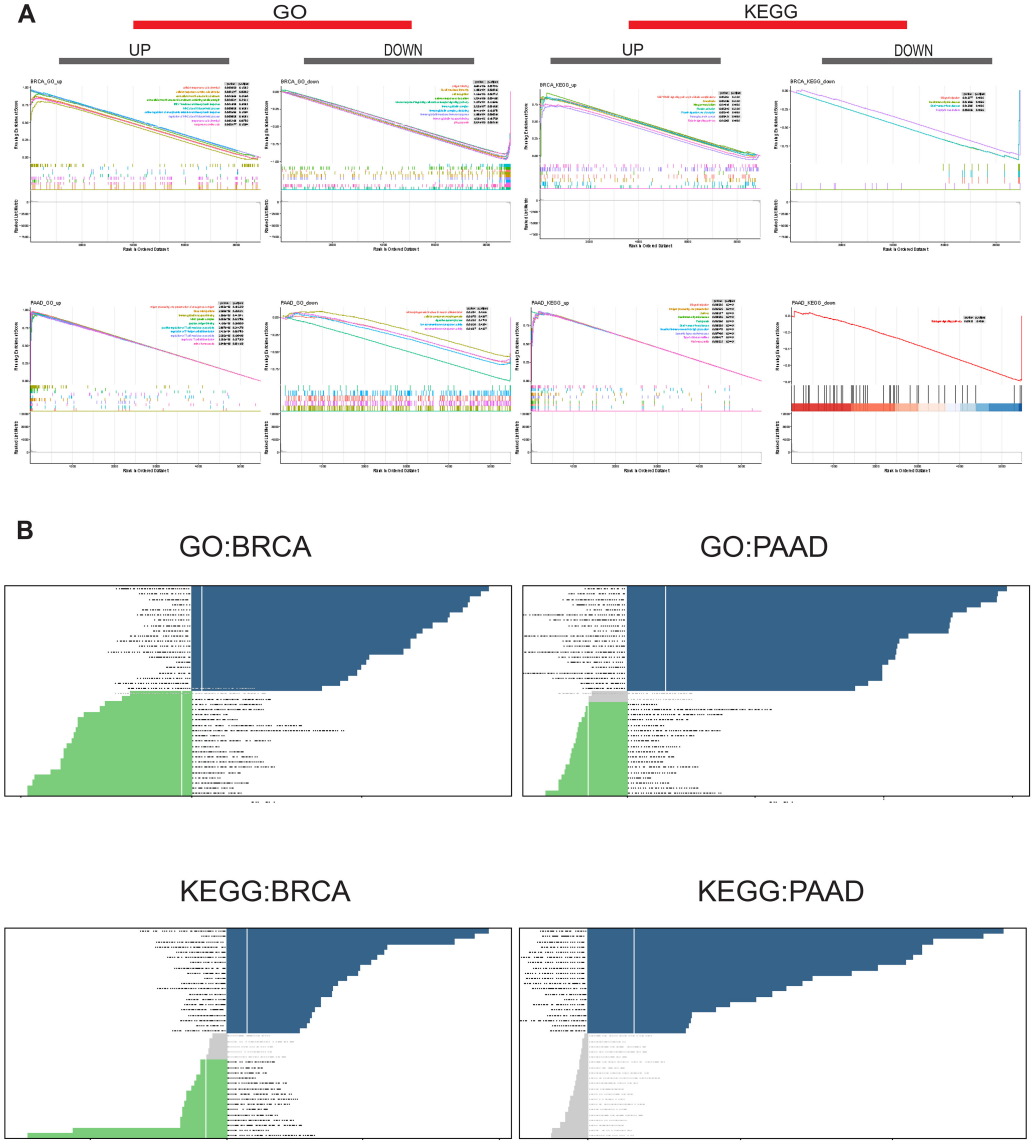
Biological implications for the expression of SUSD3 in tumors. **(A)** GO functional annotation and KEGG pathway analysis of SUSD3GSEA in BRCA and PAAD. The different colored curves depict the different functions or pathways of regulation in different types of cancer, with peaks on the ascending curve indicating positive regulation and peaks on the descending curve indicating negative regulation. **(B)** GSVA results were obtained using GO and KEGG datasets for BRCA and PAAD. The dark blue bars indicate the paths with the most significant positive correlation, the green bars indicate the paths with the most significant negative correlation, and the gray bars indicate the unimportant paths (FDR>0.05). The horizontal axis represents the log10 (P-value) of the GSVA score.

To gain deeper insights into the mechanistic role of SUSD3 in tumors, its functional pathways were further investigated through sequence function enrichment analyses. Based on the median expression levels of SUSD3 across various cancer types, the samples were stratified into two groups: one representing high expression and the other low expression of SUSD3. GSEA and GSVA were subsequently performed on both groups. In the GSEA enrichment analysis, key functional pathways were explored, focusing on the Kyoto Encyclopedia of Genes and Genomes (KEGG) and Gene Ontology (GO) categories. Notably, SUSD3 was identified as a positive regulator of several critical processes in BRCA, including extracellular matrix structural components, IRE1-mediated unfolded protein responses, platelet activation, amoebiasis, nitrogen metabolism, protein digestion and absorption, and the synthesis of proteoglycans that contribute to the tensile strength of the extracellular matrix. In contrast, SUSD3 was found to negatively regulate pathways such as circulating immunoglobulin complexes, immunoglobulin receptor interactions, and autoimmune thyroid disease. In PAAD, SUSD3 functioned as a positive regulator of immunoglobulin receptor binding, MHC protein complex formation, and systemic lupus erythematosus. However, it was also found to negatively regulate cellular processes such as cell component morphogenesis and ion transmembrane transporter activity (**Figure 10A**). These findings suggest that SUSD3 plays a complex and context-dependent role in modulating immune-related activities in BRCA and PAAD. To further comprehend the biological significance of SUSD3 expression in tumors, GSVA was conducted to assess the enrichment of specific pathways associated with its expression. The results revealed that SUSD3 expression positively correlated with numerous immune-related pathways, including immune response, inflammatory response, negative T cell selection, leukocyte-mediated signaling, immune trafficking, biosynthesis, and primary immunodeficiency pathways. Conversely, SUSD3 expression was negatively correlated with the biosynthesis of glycosphingolipids, olfactory signaling, chloride ion transport, nerve fiber junctions and synapse formation, as well as nitrogen transport and utilization efficiency (**Figure 10B**). These observations further underscore the multifaceted role of SUSD3 in modulating immune functions and cellular processes within the TME.

### 3.9 The sensitivity of SUSD3 to drugs and the relationship between molecular docking of targeted compounds

**Figure 11:**
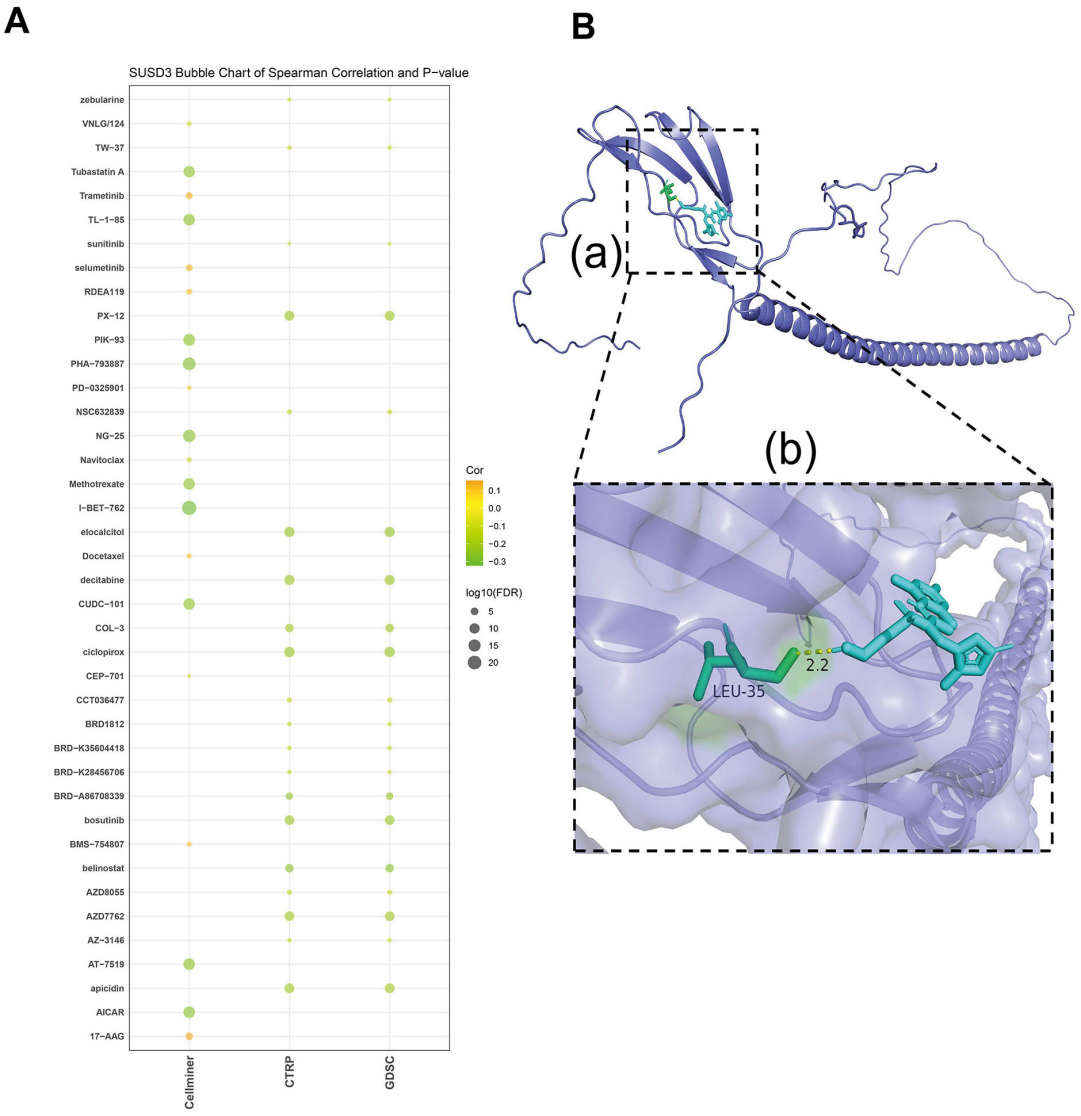
The sensitivity of SUSD3 to drugs and the relationship between molecular docking of targeted compounds. **(A)** Analyze the relationship between SUSD3 expression and predicted drug response in CellMiner, CTRP, and GDSC databases. **(B)** Prediction of Selumetinib interaction with SUSD3 protein. **(a)** A structural diagram of SUSD3 egg white versus Selumetinib is shown. **(b)** Enlarged view of the interaction of Selumetinib with SUSD3 protein.

To investigate the relationship between SUSD3 expression and potential therapeutic agents, a comprehensive drug analysis was conducted. The results revealed that several anticancer drugs, including trametinib, selumetinib, RDEA119, PD-0325901, docetaxel, BMS-754807, and 17-AAG, were positively correlated with SUSD3 expression. Conversely, drugs negatively correlated with SUSD3 expression encompassed AICAR, apicidin, AT−7519, AZD7762, belinostat, bosutinib, BRD−A86708339, ciclopirox olamine, COL−3, CUDC−101, decitabine, elocalciferol, I−BET−762, methotrexate, NG−25, PHA−793887, PIK−93, PX−12, and TL−1−85 (**Figure 11A**). To further elucidate the interaction between SUSD3 and these drugs, molecular docking studies were performed using Autodock4 software, which provided insights into the binding affinity between the SUSD3 protein and the selected anticancer drugs. The docking analysis revealed the binding sites and the maximum binding energy for each drug-SUSD3 interaction. Notably, previous research has established that selumetinib, a non-ATP-competitive and highly selective MEK1/2 inhibitor, effectively suppresses tumor cell proliferation *in vitro* (34, 35). It has been demonstrated to inhibit the proliferation of malignant peripheral nerve sheath tumor (MPNST) cells, promote apoptosis, and significantly reduce cell invasion and migration. Selumetinib exerts its antitumor effects by modulating key protein kinases in immune-related pathways (36). Molecular docking analysis of selumetinib with SUSD3 revealed not only a strong interaction between the two but also the formation of a stable structure through hydrogen bonding. The maximum binding energy between SUSD3 and selumetinib was calculated to be −2.17 kJ/mol, indicating a strong affinity between the protein and the drug (**Figure 11B**). These findings suggest that SUSD3 may serve as a potential biomarker for predicting the efficacy of specific anticancer agents.

### 3.10 Relationship between silencing of SUSD3 and cell reproduction

**Figure 12:**
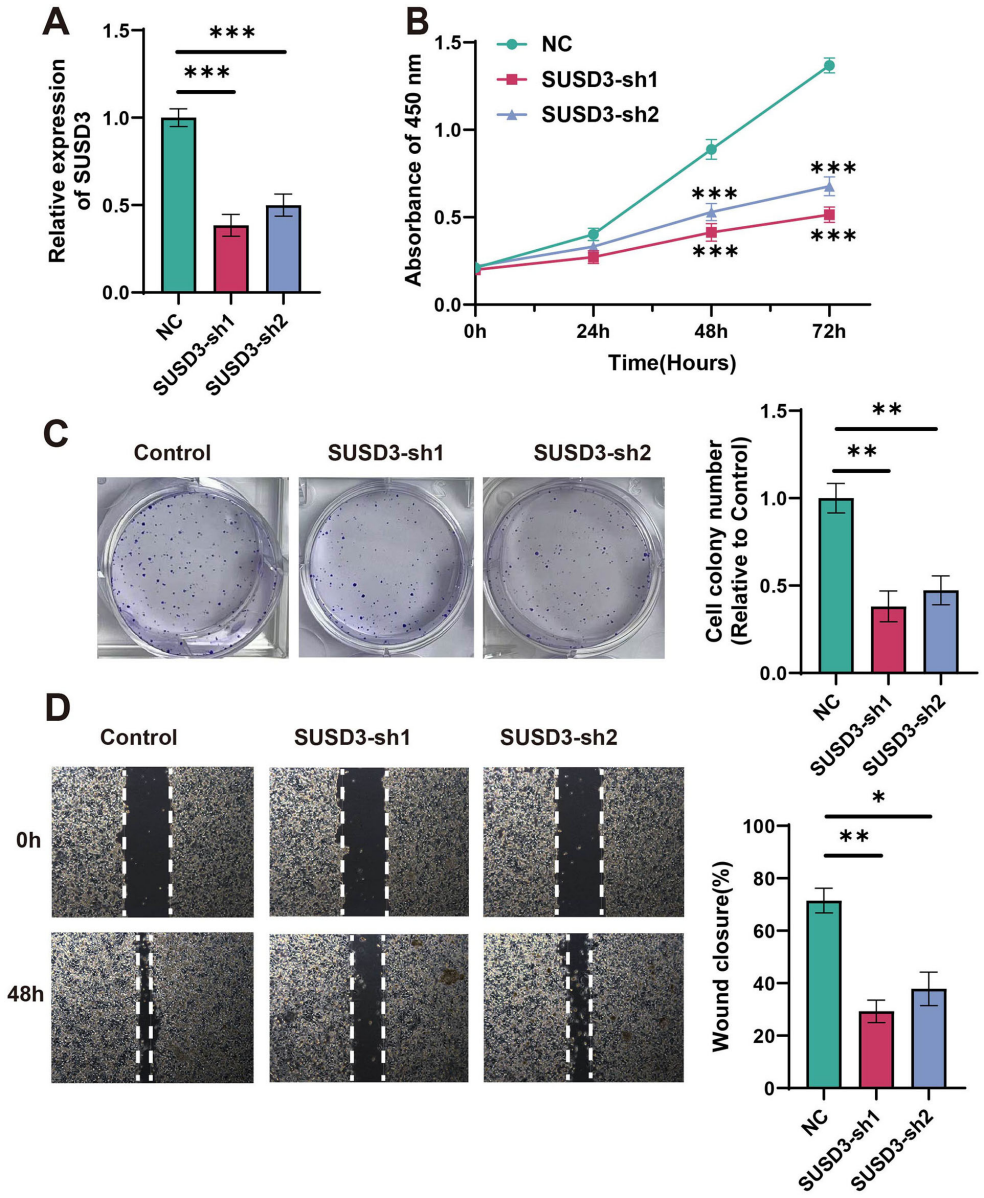
Relationship between silencing of SUSD3 and cell reproduction. **(A)** The relative expression of SUSD3 in NC, SUSD3-sh1, and SUSD3-sh2 groups after knockdown was performed. (Statistical significance was marked as ***p < 0.001). **(B)** In the CCK-8 assay results, the knockdown of SUSD3 has a significant effect on the inhibition of breast cancer cells. (Statistical significance was marked as ***p < 0.001). **(C)** The reference cell line was compared to the clone formation in SUSD3-sh1 and SUSD3-sh2. (Statistical significance was marked as **p < 0.01). **(D)** Wound healing experiments were performed on three groups of cancer cells: NC, SUSD3-sh1, and SUSD3-sh2. (mark to *p < 0.05, **p < 0.01).

Using siRNA technology, cancer cell lines with SUSD3 knockdown were generated to investigate the biological significance of SUSD3 and its underlying mechanisms. The samples were divided into three groups: one as a negative control (NC), and the other two as SUSD3-knockdown groups, namely SUSD3-sh1 and SUSD3-sh2. Compared to the NC group, the expression in the latter two groups was markedly downregulated (**Figure 12A**). The results of our experiments demonstrated a marked reduction in breast cancer cell proliferation following SUSD3 silencing (**Figures 12B, C**). Furthermore, subsequent assays revealed a significant decrease in the migratory capacity of cancer cells upon reduction of SUSD3 expression (**Figure 12D**). These findings suggest that SUSD3 plays a critical role in promoting both the proliferation and migration of cancer cells, underscoring its potential as a therapeutic target in cancer treatment.

## 4 Discussion

Cancer immunotherapy, particularly targeting immune checkpoint inhibitors, has demonstrated remarkable clinical success in treating various tumor types. However, due to tumor heterogeneity and individual variations in immune responses, the efficacy of immunotherapy remains significantly limited in numerous malignancies, presenting a substantial challenge for cancer patients (37). The identification of reliable biomarkers capable of accurately predicting patient responses to immunotherapy is essential for tailoring personalized treatment strategies. In this context, the present study highlights SUSD3 as a promising pan-cancer prognostic biomarker with the potential to predict responses to immunotherapy effectively. Moreover, the findings of this research offer valuable insights into the role of SUSD3 in cancer treatment, paving the way for further investigation into its therapeutic potential.

In recent years, the increasing incidence of cancer, particularly in women’s health, has garnered significant attention within the biomedical field. One prominent risk factor for breast cancer is the accumulation and overexposure to estrogen. AIs have emerged as the most effective endocrine treatment for estrogen-dependent cancers post-menopause. However, resistance to AIs remains the primary cause of therapeutic failure (13). Consequently, numerous studies have indicated that SUSD3 could serve as a novel tumor marker, capable of predicting therapeutic outcomes and playing a pivotal role in forecasting both the prognosis of breast cancer and the efficacy of adjuvant therapies. As research progresses, the significance of SUSD3 in various cancer types has become increasingly apparent, positioning it as a key player in the evolving landscape of pan-cancer research.

Initially, the OpenTarget tool was utilized to explore diseases associated with SUSD3, followed by an investigation of its expression across various cancer types using the TIMER database. The findings revealed a significant upregulation of SUSD3 expression in BRCA and LGG. However, high levels of SUSD3 expression were not limited to these two cancers; it was also notably elevated in five other cancer types. This suggests that SUSD3 plays distinct roles in different malignancies. Analysis of tissue samples through the Kruskal-Wallis plot further demonstrated elevated SUSD3 expression in organs such as the breast, testis, pleura, bone marrow, and lymphoid organs. According to the TISCH database, SUSD3 was predominantly expressed in immune cells, particularly monocytes/macrophages and CD4+ T cells, highlighting its potential involvement in the immune landscape of tumors.

Tumor-associated macrophages (TAMs) are key components of the TME, linked to distant metastasis in breast cancer and neuroblastoma. Targeting TAMs has shown potential for improving prognosis, suggesting that SUSD3 may influence cancer outcomes by modulating TAMs and other immune components (38, 39).

Next, the correlation between SUSD3 expression and various prognostic outcomes, including OS, DSS, DFI, and PFI, was assessed. The results were highly consistent across these endpoints, demonstrating that SUSD3 is significantly associated with the prognosis of cancer patients. Notably, while SUSD3 expression showed no correlation with the prognosis of KIRC, it was strongly linked to the prognosis of most other cancer types. These findings underscore the critical role of SUSD3 in cancer prognosis and suggest that it may serve as a potent prognostic biomarker with the potential to guide clinical outcomes in cancer patients.

Subsequently, the cBioPortal database was utilized to investigate whether the SUSD3 gene undergoes alterations at the genomic level across different cancers. Notably, in the majority of DLBC, SUSD3 was found to be extensively deleted. Further analysis of the relationship between CNV, DNA methylation, and SUSD3 gene expression revealed a significant positive correlation between CNV, DNA methylation, and SUSD3 expression, except in LGG and UCEC, where a negative correlation was observed. In addition, it was observed that patients with ACC exhibited poor prognosis across all three survival metrics. Interestingly, higher levels of SUSD3 methylation were associated with a decreased risk of death in these patients, suggesting a protective role of SUSD3 methylation in ACC prognosis.

In the era of ultra-precise medical care, the prominence of immunotherapy is rapidly expanding, and the use of the TMB as a prognostic marker is increasingly recognized as a valuable tool for predicting immunotherapy responses in cancer patients (37, 40). Additionally, MSI has emerged as an important biomarker for predicting responses to anti-PD-1 inhibitors (41). Our analysis revealed a positive correlation between SUSD3 expression and TMB in KIRP and BLCA, as well as a positive correlation between SUSD3 expression and MSI in BLCA. These findings suggest that modulating SUSD3 levels could influence TMB and MSI, further underscoring the critical role of SUSD3 in predicting immunotherapy efficacy. Moreover, by examining cohorts of cancer patients undergoing ICI therapy, particularly those receiving anti-PD-1 and anti-PD-L1 treatments, it was observed that patients with high SUSD3 expression had significantly better survival rates and longer response durations compared to those with low SUSD3 expression. These results collectively confirm that SUSD3 could serve as a reliable biomarker for predicting responses to immune checkpoint blockade therapy, positioning it as a promising marker for personalized cancer immunotherapy.

A critical role in the tumor microenvironment is played by tumor-infiltrating immune cells, which can promote or inhibit tumor development and progression (42). In light of this, the relationship between SUSD3 expression and immune cell infiltration was investigated. The results revealed a strong correlation between SUSD3 expression and immune cell infiltration. In the majority of tumor types, SUSD3 expression was positively correlated with the infiltration of B cells, CAFs, dendritic cells, CD4+ T cells, macrophages, CD8+ T cells, monocytes, NK cells, and Treg cells. This suggests that SUSD3 expression may influence cancer prognosis and progression by modulating the TME. Previous studies have shown that TAMs in the TME are associated with migration and prognosis in breast cancer (38). Therefore, controlling macrophage populations could provide a means to elucidate the role of SUSD3 in breast cancer patients, potentially altering prognostic outcomes.

In our study, we confirmed that SUSD3 is closely associated with immune cells and related molecules across most cancer types. With the exception of BRCA, KICH, and KIRC, SUSD3 exhibited a positive correlation with immune-related genes in all other cancers, including MHC proteins, immunosuppressive factors, immune-activating proteins, chemokine receptors, and chemokines. These results further underscore the strong relationship between SUSD3 expression and immune infiltration in tumor cells, offering new insights and potential therapeutic targets for cancer treatment.

TME is highly complex and plays a crucial role in tumor progression, metastasis, and therapeutic resistance. Current strategies focus on exploring various interventions, including physical methods and active exercise, to modulate the TME and improve treatment outcomes (43, 44). The high expression of SUSD3 in immune cells within the TME suggests that it may serve as a potential therapeutic target capable of influencing the cancer immune response.

GSEA results revealed that SUSD3 is closely linked to immune activation processes, acting as a positive regulator of several key pathways, including IRE1-mediated unfolded protein responses, amebiasis, and proteoglycan pathways in cancer. Further investigation through GSVA confirmed the association between SUSD3 and various immune-related and immune factor-related pathways across different cancer types, including immune response, inflammatory response, negative T cell selection, leukocyte-mediated signaling, trafficking, biosynthesis, and primary immunodeficiency, among others. Additionally, differentially expressed genes (DEGs) that are co-expressed with SUSD3 have been shown to be significantly involved in multiple biological processes such as cell cycle regulation, DNA replication, p53 signaling, cancer-associated pathways, and Wnt signaling pathways (45). These findings suggest that SUSD3 may influence the tumor immune microenvironment by modulating these molecular pathways, offering valuable new insights into its role in cancer biology. These results underscore the potential of SUSD3 as a key player in cancer immunology and highlight its promise as a target for future cancer immunotherapy strategies. Understanding the precise mechanisms through which SUSD3 interacts with immune and cancer-related pathways could pave the way for novel therapeutic approaches, particularly those aiming to manipulate the immune environment for enhanced cancer treatment outcomes.

In the search for potential drugs that interact with SUSD3, a comprehensive screening was conducted using databases such as CellMiner, CTRP, and GDSC. This analysis identified several anticancer drugs positively correlated with SUSD3 expression, including trametinib, selumetinib, RDEA119, PD−0325901, docetaxel, BMS−754807, and 17−AAG. Notably, selumetinib, a non-ATP-competitive and highly selective MEK1/2 inhibitor, has previously been shown to effectively inhibit tumor cell proliferation in various *in vitro* studies (14, 15). It does so by promoting apoptosis and inhibiting cell invasion and migration in MPNST cells. The mechanism by which selumetinib suppresses tumor growth is primarily through regulating key protein kinases involved in immune-related pathways (46). In addition to selumetinib, other studies have also highlighted a positive correlation between SUSD3 and the efficacy of other therapeutic agents, such as fulvestrant, raloxifene, and flufenazine (45). These findings suggest that targeting SUSD3 might enhance the effectiveness of these drugs in treating certain cancers. The hypothesis that selumetinib can specifically target and inhibit SUSD3 protein aligns with these observations, providing a promising new therapeutic avenue. This insight suggests that incorporating SUSD3 as a biomarker or therapeutic target could open up new possibilities in cancer treatment, particularly in combination with existing MEK1/2 inhibitors and other targeted therapies. Overall, these results not only underscore the potential role of SUSD3 in cancer treatment but also highlight selumetinib as a possible candidate for further clinical evaluation, possibly through its effects on the SUSD3-related pathways. Further studies to validate this hypothesis could provide the necessary groundwork for developing more effective, personalized cancer therapies. In the future, research methods such as those employed by Asma Mokashi et al. could be referenced to integrate databases like PubChem, BindingDB, UniProt, and DisGeNET to expand the drug sample library. This approach could facilitate further analysis and exploration of other potential drugs targeting SUSD3 (47).

In this study, SUSD3 was knocked down in MCF-7 cells to observe its effects on the proliferation and migration of breast cancer cells. The results indicate that SUSD3 represents a promising target for the development of anti-BRCA therapies. Previous studies have demonstrated that low-frequency rotating magnetic fields can inhibit breast cancer metastasis through modulation of F-actin, with minimal impact on normal cells (48). This suggests that there are diverse approaches to inhibiting breast cancer migration. To ensure the accuracy of these findings, further *in vitro* studies are necessary to strengthen the conclusions and enhance their clinical relevance.

In conclusion, this comprehensive pan-cancer analysis of SUSD3 has highlighted its potential as both a cancer prognostic biomarker and a predictor of immunotherapy response. The study established clear correlations between SUSD3 expression and various key cancer characteristics, including prognosis, immune regulation, immune cell infiltration, tumor microenvironment, TMB, and MSI. These findings suggest that SUSD3 plays a significant role in modulating immune responses and the overall tumor progression across multiple cancer types. Notably, SUSD3 has shown strong potential as a prognostic marker in breast cancer, where its high expression correlates with poor prognosis, emphasizing its importance in the clinic (49). The association between SUSD3 expression and immune factors further supports its role in influencing cancer immunotherapy outcomes, making it a promising candidate for predicting patients’ responses to immunotherapies such as ICIs. However, while this study offers valuable insights into SUSD3’s prognostic and predictive potential, the specific molecular mechanisms by which SUSD3 influences cancer progression and therapy response are still not fully understood. More in-depth experimental studies are required to better elucidate these mechanisms and validate SUSD3 as a reliable biomarker for clinical use. As research into SUSD3 continues to advance, it holds the potential to significantly enhance personalized cancer treatment strategies and help in the development of new therapeutic approaches targeting its related pathways. The promise of SUSD3 as both a predictive biomarker and a target for therapy could offer novel, effective solutions for cancer treatment and prognosis, ultimately improving patient outcomes.

## Data Availability

Requests to access the datasets should be directed to SW, wangshiyan@hyit.edu.cn.
